# The Association of Vitamin D Levels with Common Pregnancy Complications

**DOI:** 10.3390/nu10070867

**Published:** 2018-07-05

**Authors:** Andraž Dovnik, Faris Mujezinović

**Affiliations:** University Clinic for Gynaecology and Perinatology, Maribor University Medical Centre, Ljubljanska 5, SI-2000 Maribor, Slovenia; fmujezinovic@gmail.com

**Keywords:** vitamin D, pregnancy outcome, nutritional supplements

## Abstract

The association between vitamin D deficiency and various adverse pregnancy outcomes has been extensively investigated in recent years. The pregnant woman is the only source of vitamin D for the foetus. The main sources of vitamin D for pregnant women are sunlight, fortified dairy products, oily fish and dietary supplements. Vitamin D deficiency during pregnancy has been associated with some adverse neonatal outcomes as well as an increased risk of late pregnancy complications. The outcomes of the published studies investigating preeclampsia and gestational diabetes mellitus vary with some large trials suggesting a potential positive effect of vitamin D supplementation during pregnancy on the decreased risk of these complications. Research also suggests a possible connection between lower vitamin D concentrations and increased risk of preterm labour. In our manuscript, we aim to review the existing literature regarding the prevalence of vitamin D deficiency during pregnancy, the factors associated with vitamin D deficiency, and possible pregnancy complications arising from it.

## 1. Introduction

The association between vitamin D deficiency and various adverse pregnancy outcomes has been extensively investigated in the past years. It has been postulated that vitamin D deficiency could be associated with increased risk of preeclampsia, gestational diabetes mellitus, caesarean section and bacterial vaginosis in pregnancy [1]. With the aim of reviewing the existing literature on this subject, the PubMed/Medline electronic database was searched for studies published in the English language up to May 2018. The Medical Subject Headings (MeSH) database was searched for the keywords ‘vitamin D’, ‘gestational diabetes’, ‘preeclampsia’, ‘preterm labour’, ‘bacterial vaginosis’ and ‘caesarean section’. In our review we aimed to include observational studies, supplementation studies and meta-analyses dealing with the effect of vitamin D on the occurrence of gestational diabetes, gestational hypertension and preeclampsia, preterm labour, bacterial vaginosis and caesarean section. 

## 2. Physiology of Vitamin D during Pregnancy 

The compound 25-hydroxyvitamin D (25(OH)D) is the circulating form of vitamin D and the one that is usually measured [2]. The active form of vitamin D is 1,25-dihidroxyivitamin D (1,25(OH)2D) which is formed by the action of the enzyme 1α-hydroxylase in the kidneys [3]. 1α-hydroxylase is also active in the placenta, dendritic cells, parathyroid gland, epidermis and bones [4,5]. Vitamin D has an important role in maintaining an adequate level of minerals through its influence on calcium and phosphate metabolism for bone mineralisation and metabolic functions [2].

The concentration of 25(OH)D is relatively constant throughout pregnancy [6]. The mother is the only source of vitamin D for the foetus [7]. Vitamin D concentrations in the umbilical cord are usually 60–89% of the values in the mother’s blood. The active form of vitamin D does not cross the placenta. However, its concentrations in the mother’s blood are doubled in pregnancy, probably because of its production in foetal tissues and placenta [1,8]. The concentration of 1,25(OH)2D is determined by the activity of the enzymes 1α-hydroxylase and 24-hydroxylase [9]. 1α-hydroxylase is a product of the gene *CYP27B1* which is expressed in the kidneys, decidua and placenta during pregnancy. On the other hand, 24-hydroxylase is an enzyme responsible for the production of less potent vitamin D metabolites and is a product of the gene *CYP24A1*. The rise in the concentration of 1,25(OH)2D during pregnancy could be due to the methylation of catabolic gene *CYP24A1*. As shown by Novakovic et al., the methylation of *CYP24A1* gene promoter could cause the decreased activity of this gene and consequently the decreased activity of 24-hydroxylase [9]. The increased concentration of 1,25(OH)2D could also be caused by increased concentrations of calcitonin during pregnancy [10]. Calcitonin increases the activity of 1α-hydroxylase and thus the production of 1,25(OH)2D irrespective of serum calcium concentration [10]. Consequently, the intestinal calcium absorption increases [1]. Despite a 100% increase in 1,25(OH)2D concentration the serum calcium concentration in the mother remains constant [11]. Another important change during pregnancy is a rise in the concentration of vitamin D-binding-protein [12]. The circulating form of vitamin D is bound to vitamin D-binding-protein which is filtered in the glomeruli of the kidneys and then reabsorbed in the proximal tubules. Vitamin D-binding-protein could have a role in the function and metabolism of vitamin D during pregnancy. It has a higher affinity to 25(OH)D in comparison to 1,25(OH)2D and thus plays an important role in maintaining 25(OH)D as it promotes the reabsorption of 25(OH)D from the glomerular filtrate [12].

The compound 1,25(OH)2D as the active form of vitamin D has non-genomic and genomic effects through its action on vitamin D receptor [13]. The non-genomic effects occur quickly and include the activation of ion channels with the change of electrical state of the cell and protein kinase activation. On the other hand, the genomic effects which include the modulation of gene expression take more time. The abnormalities of the action of vitamin D receptor could manifest in signs and symptoms of vitamin D deficiency. During pregnancy, this may present as gestational diabetes, preeclampsia, preterm birth or miscarriage in early stages of pregnancy [13].

The main sources of vitamin D are sunlight, oily fish, fortified dietary products and nutritional supplements [2]. Vitamin D supplements are available as prescriptions and as over the counter preparations. As prescriptions, capsules of vitamin D_2_ (ergocalciferol) are available that contain 50,000 IU of vitamin D_2_ per capsule. The other prescription product is a liquid form which contains 8000 IU/mL of vitamin D_2_. Multivitamin preparations are available over the counter and contain 400 IU of vitamin D_2_ or D_3_. Vitamin D_3_ in products containing 400 IU, 800 IU, 1000 IU and 2000 IU is also available over the counter [2]. The foods with the highest vitamin D content are fish oil (400–1000 IU/spoon of oil), caught salmon (600–1000 IU/100 g), eel (1200 IU/100 g), herring in oil (400–800 IU/100 g), sardines (300 IU/100 g), salmon in a tin can (300–600 IU/100 g), herring in a tin can (250 IU/100 g), tuna in a tin can (230 IU/100 g), shiitake mushrooms (100 IU/100 g), egg yolk (20–50 IU/1 egg yolk), cow milk (0.4–1.2 IU/100 mL), mothers milk (1.5–8 IU/100 mL) and cheese (7–28 IU/100 g) [14].

Many studies have shown a positive effect of the intake of nutritional supplements on vitamin D concentration in pregnant women [15,16,17,18,19]. However, these studies were very heterogeneous in number and methodology. Hollis et al. compared three different dosages of supplemented vitamin D, namely 400 IU, 2000 IU and 4000 IU daily [20]. The group which received 4000 IU of vitamin D reached the highest concentrations [20]. In a subsequent analysis of this randomized controlled trial, the authors suggested the supplementation dose of 4000 IU of vitamin D as the one which enables pregnant women to achieve optimal vitamin D concentrations [21]. This was the first paper to report in an intention-to-treat fashion that supplemented vitamin D decreased some important pregnancy complications. They found that the group of women who received 4000 IU daily vitamin D supplementation during pregnancy had an average concentration of 111.0 nmol/L 25(OH)D at the time of birth. In this group, the incidence of several pregnancy complications such as preeclampsia, gestational diabetes, preterm labour, caesarean section and infection was the lowest [21]. In addition, a recent Iranian trial has shown that more than one half of participants achieved sufficient vitamin D concentrations with vitamin D supplementation in comparison to only 2% of women who did not receive vitamin D supplements [22]. On the other hand, other studies failed to prove an increase in vitamin D concentration with supplementation [23]. A recent meta-analysis by Palacios et al. confirmed higher vitamin D concentrations when vitamin D was supplemented during pregnancy [24,25].

The principal source of vitamin D in certain populations is sunlight [12,26]. Factors which negatively influence vitamin D production in the skin are living mostly in an indoor environment, covering the skin with clothes, avoiding sunlight by staying in the shade and using sunscreen, sun exposure through glass, and air pollution with increased ozone concentration which absorbs ultraviolet light [12]. Black skin is 90% less capable of vitamin D production, and sunscreen decreases vitamin D production in the skin by 95–99% [27]. Vitamin D production is also decreased with higher geographical latitude [12].

Various researchers have studied the influence of season and sunlight exposure on vitamin D concentrations in pregnancy. The average daily exposure to sunlight was higher in women with higher vitamin D concentrations [16]. Higher average monthly temperatures also contributed to higher vitamin D concentrations with significant differences in vitamin D concentrations in different seasons of the year [17,28,29,30].

Only a few studies have investigated the correlation between various measured meteorological parameters and vitamin D concentrations in pregnant women [31,32]. Our own research focused on the relationship between the average temperatures and the duration of sunlight in the 30 days preceding delivery and vitamin D concentrations in Central European pregnant women. A highly positive correlation between these meteorological parameters and vitamin D concentrations in pregnant women at term was observed, independent of factors associated with nutrition and lifestyle [31]. This study showed that despite the lack of data about lifestyle during pregnancy the measured meteorological data could be useful in assessing the environmental influence on vitamin D concentration.

## 3. Gestational Diabetes Mellitus

Gestational diabetes is defined as glucose intolerance which is first diagnosed in pregnancy. The incidence of gestational diabetes is 2–6% in Europe [33] and around 14% in the US [34]. 

There are many ways through which vitamin D could affect glucose metabolism. Pancreatic β-cells express 1α-hydroxylase; the active form of vitamin D binds on vitamin D receptor on pancreatic β-cells; the vitamin D response element is present in the human insulin gene promoter [35]. There is also some evidence about the role of vitamin D in maintaining glucose tolerance through its influence on insulin secretion and sensitivity [36].

Many research groups have investigated the connection between vitamin D deficiency and the incidence of gestational diabetes and have reported diverse outcomes. Some observational studies found a higher risk of developing gestational diabetes with lower vitamin D concentrations [37,38,39,40,41,42,43,44] while other observational studies failed to prove this association [45,46,47,48]. It is likely that gestational diabetes is influenced by vitamin D levels in early pregnancy since it is most commonly diagnosed in the second and third trimester [49]. A prospective study compared vitamin D levels at around 16 weeks of pregnancy in 57 women who later developed gestational diabetes and 114 controls who did not develop gestational diabetes [37]. The majority of participants were Caucasian/non-Hispanic. Vitamin D levels were lower in women who developed gestational diabetes even after controlling for the known risk factors for gestational diabetes, such as body mass index, race, age and family history of type 2 diabetes [37]. Similarly, a study on 655 pregnant women in Canada, 97% of whom were of European descent, showed that lower concentrations of vitamin D in the first trimester are a risk factor for developing gestational diabetes [39]. It has also been postulated that deficient vitamin D status in women with gestational diabetes increases the risk of poor neonatal outcomes such as neonatal hypoglycaemia and small for gestational age [50].

Several meta-analyses of observational studies have been performed on this topic and they all reported lower vitamin D concentrations in women with gestational diabetes [51,52,53,54,55]. The number of observational studies included in these meta-analyses ranged from seven [51] to twenty-four [52]. Vitamin D concentrations below 50 nmol/L were associated with an increased risk of gestational diabetes with odds ratios between 1.38 [52] and 1.6 [51]. The latest meta-analysis of 29 studies was performed by Hu et al. [36]. The vitamin D cut-off values in the included observational studies were in the range of 25–75 nmol/L, but most studies used the cut-off value 50 nmol/L. The results indicated that vitamin D insufficiency increased the risk of gestational diabetes by 39%. The average level of vitamin D in women with gestational diabetes was 4.78 nmol/L lower than in pregnant women without gestational diabetes [36].

The effect of vitamin D supplementation throughout pregnancy on the incidence of gestational diabetes has been extensively investigated. Yap et al. studied the influence of various quantities of supplemented vitamin D on the occurrence of impaired glucose tolerance in Australian women of mixed ethnic background [56]. The study included 179 pregnant women with vitamin D concentrations below 80 nmol/L before the 20th week of pregnancy who were randomised into two groups. One group received 5000 IU of daily vitamin D supplements and the other 400 IU. They found no difference in the incidence of impaired glucose tolerance between the 26th and the 28th week of pregnancy [56]. American researchers performed a pooled analysis of two prospective randomized trials with 504 participants of mixed races [57]. Pregnant women were enrolled in the study before the 16th week of pregnancy. Three different supplementation regimens were compared. The first group received 400 IU of vitamin D daily, the second 2000 IU and the third 4000 IU. The incidence of gestational diabetes in the three groups was not significantly different, but there was a trend of lower incidence in the group which received higher vitamin D dosages [57]. In a study on 133 Chinese pregnant women with diagnosed gestational diabetes, authors aimed to observe the effect of different doses of supplemented vitamin D on insulin resistance [58]. The control group received no vitamin D supplementation, 200 IU daily of vitamin D was supplemented in the low dosage group, 50,000 IU monthly in the medium dosage and 50,000 IU every two weeks in the high dosage group. The authors concluded that high amounts of supplemented vitamin D increased insulin responsiveness and lowered insulin levels in women with gestational diabetes which could be beneficial in lowering maternal and neonatal complications related to gestational diabetes [58].

The controversial findings of these observational and supplementation studies implicate that gestational diabetes is a multifactorial disease influenced by risk factors such as BMI, lifestyle and weight gain. Vitamin D deficiency is more prevalent in obese women who also have a higher incidence of gestational diabetes, which makes the analysis of the influence of vitamin D on the occurrence of gestational diabetes even more difficult [47]. Furthermore, the cross-sectional nature of these studies represents a major limitation since the temporal relationship between measured vitamin D and the risk of developing gestational diabetes is not clear [53]. 

Based on the existing literature it is probable that vitamin D deficiency could be one of the cofactors that increase the risk of gestational diabetes. Using Hill’s criteria for interpreting an association as causative, one could argue that the evidence in favour of a causative association between vitamin D deficiency and gestational diabetes is moderately strong [59]. Several meta-analyses of observational studies indicated an 18–80% increased risk of gestational diabetes in women with vitamin D deficiency [36,54,55,60]. Furthermore, the data is fairly consistent as the association has been shown in different places and in patients of different ethnical origins [59]. However, the most recent meta-analysis showed a statistically significant association between low vitamin D levels and gestational diabetes only in developing countries and not in developed countries [60]. Regarding other Hill aspects of cause and effect, namely specificity, temporality, biological gradient, plausibility, coherence, and experiment, the evidence is inconsistent [59]. It is our opinion that vitamin D should be supplemented in pregnant women since supplementation has been clearly shown to be safe and it has a potential of lowering the incidence of gestational diabetes. We believe that national recommendations regarding vitamin D supplementation should be followed where these are in place and implemented where vitamin D supplementation is not yet recommended. 

## 4. Gestational Hypertension and Preeclampsia

The exact mechanism of altered vitamin D metabolism in patients with preeclampsia and hypertensive disorders is not fully understood [61].

Many observational studies found a significant association between lower vitamin D concentrations and higher risk of preeclampsia. A comparative study in mixed ethnicity American pregnant women has been performed by Baker et al [62]. Vitamin D values were measured between the 15th and the 20th week of pregnancy in 51 women who later developed severe preeclampsia were compared to vitamin D values in 204 women with uncomplicated pregnancies. The groups were matched by ethnicity. The median vitamin D concentrations in women who later developed preeclampsia were 23% lower compared to the control group. Higher incidence of vitamin D deficiency was observed in the study group [62]. In a similar number of mixed ethnicity American women, lower vitamin D concentrations before the 16th week of pregnancy were observed in women who later developed preeclampsia in a study by Bodnar et al. [63]. The average vitamin D concentration in the preeclampsia group was 45.4 nmol/L compared to 53.1 nmol/L in the control group. The difference was statistically significant [63]. Unlike the study group from the paper by Baker et al. which consisted of women with severe preeclampsia, most women included in the study group by Bodnar et al. had mild preeclampsia [62,63]. Other investigators also reported lower vitamin D concentrations in women with preeclampsia or those who developed preeclampsia later in pregnancy in comparison to those who did not [52,64,65,66]. On the other hand, some observational studies found no difference in vitamin D concentrations in women with preeclampsia compared to normotensive women. A study in 221 mixed ethnicity Canadian women measured vitamin D concentration between the 15th and the 20th week of pregnancy [67]. The enrolled women were divided into three groups according to their vitamin D status: below 37.5 nmol/L, between 37.5 nmol/L and 75 nmol/L, and above 75 nmol/L. They found no difference among the three groups in the occurrence of preeclampsia later in pregnancy. However, this result could be influenced by the relatively small number of participants as only 28 patients developed preeclampsia [67]. Other authors also reported that vitamin D concentration in the first trimester did not influence later development of preeclampsia [68,69]. Postpartum vitamin D concentrations did not differ among 44 women with preeclampsia and 54 normotensive women in a study by Dalmar et al. [70].

In a meta-analysis of four observational studies, Harvey et al. found no increased risk of preeclampsia in women with lower vitamin D concentrations [71]. In contrast, a Canadian meta-analysis of 31 observational studies found a higher risk of preeclampsia in vitamin D concentrations lower than 50 nmol/L [72]. A recent systematic review of observational, interventional and dietary studies supported these findings [61]. Wei et al. analysed 24 observational studies and also found an increased risk of preeclampsia in women with vitamin D concentrations below 50 nmol/L [52].

A meta-analysis of two studies of vitamin D supplementation showed a trend toward a reduction in preeclampsia with supplementation, but this did not reach statistical significance [24]. The daily doses of vitamin D in this meta-analysis ranged from 200 IU to 2000 IU, weekly doses ranged from 35,000 IU to 120,000 IU, and monthly doses ranged from 200,000 IU to 600,000 IU [24]. In addition, the same meta-analysis analysed three trials evaluating the role of combined supplementation with vitamin D and calcium and showed a significantly reduced risk of preeclampsia in the group of women receiving supplementation [24]. Another recent meta-analysis of 27 randomized controlled trials showed that vitamin D supplementation could reduce the risk of preeclampsia by 57% and calcium supplementation could reduce the risk by 51% [73]. In addition, a Cochrane systematic review showed that calcium supplementation decreased the risk of preeclampsia especially in women who had higher risk of preeclampsia and had lower calcium diets [74].

The interpretation of the published observational studies is limited by their variable definition of preeclampsia, discrepancies in the analysis of confounding factors and differences in the gestational age at which vitamin D concentration was assessed. A low number of patients with preeclampsia was included in most of the studies [71]. Among other variables, vitamin D status is influenced by the gestational age, obesity and ethnicity, factors that can affect the risk of preeclampsia as well. Therefore, it is more likely that the risk of preeclampsia is increased in a certain range of vitamin D concentrations and not below a certain threshold [61]. 

When evaluating the association between vitamin D deficiency and preeclampsia, there are on one hand the meta-analyses of observational studies which have shown a positive association [52,72] and on the other hand the systematic review of randomised controlled trials which has not shown any effect of vitamin D supplementation on the prevention of preeclampsia [25]. The strength of the association is similar to that of the association of low vitamin D with gestational diabetes, with the reported ORs in meta analyses in the range of 1.79 to 2.09 [52,72], but there is less consistency in the available evidence [59]. Other Hill criteria are not met [59]. To date we have robust evidence that calcium supplementation decreases the risk of preeclampsia [73,74]. The question which still needs to be answered is whether the combined supplementation of calcium and vitamin D is as effective as calcium alone in lowering the risk of preeclampsia. Calcium supplementation should be given to high risk women with low calcium diet. Given the available literature, vitamin D supplementation could be recommended to pregnant women as vitamin D is responsible for calcium homeostasis and helps to maintain adequate calcium levels which are inversely associated with blood pressure [73]. 

## 5. Preterm Labour

Vitamin D could influence the pathophysiology of preterm labour as it affects the processes of inflammation and immunomodulation [75]. It is responsible for an adequate function of toll-like receptors which initiate the innate immune response. The susceptibility to infection is increased in cases of vitamin D deficiency because of impairment of toll-like mediated induction of antimicrobial peptide cathelicidin from macrophages [76]. 

Several observational studies found no association between preterm labour and maternal vitamin D levels [67,68,77,78,79,80]. Baker et al. compared vitamin D levels in 120 American mixed ethnicity women who delivered at term and 40 women who delivered between the 23rd and the 35th week of pregnancy [81]. No differences in vitamin D levels between the groups were observed [81]. In contrast, Bodnar et al. analysed a group of twin pregnancies in mixed ethnicity American pregnant women and found significantly lower levels of vitamin D in 75 women who delivered before the 35^th^ week compared to 136 who delivered after the 35th week of pregnancy. Women with vitamin D concentrations lower than 75 nmol/L delivered prematurely in 49.4% compared to 26.2% of preterm deliveries in women with vitamin D concentrations higher than 75 nmol/L [75]. The results of some these studies need to be interpreted with caution as some of them focused on specific populations such as mothers with previous history of preterm birth [77], twin gestations [75] and women with higher risk of preeclampsia [63] and their results might not be applicable to general mixed ethnicity population such as the population in the United States [76]. Furthermore, the study by Baker et al. included more than 50% of Caucasian pregnant women and vitamin D deficiency is more prevalent in the black population. Another limiting factor in this analysis was the low rate of preterm birth at 2.8% [81]. A meta-analysis of seven observational studies failed to prove an association between maternal vitamin D levels and preterm birth [71]. In observational studies, vitamin D concentration was measured in different stages of pregnancy, not all studies were adjusted for confounders and the definition of preterm labour was not consistent between the studies. It is probably due to this variability that this meta-analysis failed to show an association.

An inverse association between vitamin D and preterm birth was reported by a recent American mixed ethnicity supplementation study [82]. Vitamin D was measured at the first prenatal visit and vitamin D supplementation offered. The participants received capsules containing 5000 IU of vitamin D according to a supplementation scheme that was personalized for each participant based on the initial vitamin D concentration by using an algorithm of existing dose-response data. Additional measurements were performed between the 24th and the 28th week of pregnancy and at the time of delivery. They reported a 62% lower risk of preterm labour in women with vitamin D concentrations higher than 40 ng/mL at the time of delivery compared to those with concentrations below 20 ng/mL. Additionally, in women who had vitamin D concentrations below 40 ng/mL at the first visit, a 60% lower risk of preterm labour was reported in those who achieved concentrations higher than 40 ng/mL on the follow up visit [82]. The major strength of this supplementation study was that the relationship between vitamin D and preterm birth was studied on a general population and it included participants with different medical conditions, socioeconomic status and different race groups. Higher vitamin D concentrations were associated with a lower risk of spontaneous preterm birth as well as preterm birth induced due to various medical conditions such as pre-existing diabetes, maternal hypertension, and previous preterm birth. Furthermore, the inverse association between vitamin D and the risk of preterm birth was found in all ethnic groups indicating a possibility that adequate vitamin D concentrations could decrease the difference in the incidence of preterm birth between ethnic groups [82]. The results of this study are in accordance with the results published by Wagner et al. who observed a 57% lower risk of preterm delivery in American mixed ethnicity women with vitamin D concentrations higher than 40 ng/mL within 6 weeks of delivery compared to women with vitamin D levels below 20 ng/mL [83]. 

As for the association of vitamin D with other pregnancy complications, the association with preterm birth is difficult to interpret because of possible confounding variables. The promising evidence from the recent interventional studies will need confirmation in further randomized controlled trials before vitamin D supplementation with the aim of reducing the risk of preterm birth can be recommended.

## 6. Bacterial Vaginosis

Regarding infections during pregnancy most of the research concentrated on the effect of vitamin D concentrations on the occurrence of bacterial vaginosis. Vitamin D induces the expression of antibacterial proteins and enhanced killing of bacteria in various tissues [84]. 

Bodnar et al. analysed a group of 469 American pregnant women that were half Caucasian and half black [85]. Vitamin D concentrations were measured before the 16th week of pregnancy simultaneously with a vaginal smear. They found that vitamin D deficiency was associated with bacterial vaginosis in black women but not in Caucasians [85]. In another American study in 146 mixed ethnicity pregnant women among whom 8.8% developed bacterial vaginosis, significantly lower concentrations of vitamin D were observed in patients with bacterial vaginosis [79]. Hensel et al. reported that vitamin D deficiency was associated with bacterial vaginosis only in pregnant and not in nonpregnant women [86]. A meta-analysis of three observational studies reported an inverse association between maternal vitamin D and the risk of bacterial vaginosis, but the thresholds used were different in each of the three studies and the possibility of additional confounding was strong in the meta-analysis [71].

Two randomized supplementation trials did not show a positive effect of vitamin D supplementation on the occurrence or recurrence of bacterial vaginosis during pregnancy [20,57].

The interpretation of the relatively small number of existing observational studies on the association of vitamin D levels and bacterial vaginosis is limited by their inhomogeneous methodology and potential confounders [71]. In addition, two randomized trials have not shown any effect of vitamin D supplementation on lowering the rates of bacterial vaginosis during pregnancy [20,57]. Therefore, it is currently not justified to supplement vitamin D in pregnancy to lower the rates of bacterial vaginosis [71].

## 7. Caesarean Section

A possible reason for the potential higher risk of caesarean delivery in women with lower vitamin D concentrations was hypothesised to be reduced pelvic muscle strength leading to prolonged labour [87].

In American pregnant women of mixed ethnicity, a significantly higher risk of caesarean section was associated with vitamin D concentrations below 37.5 nmol/L in a study by Merewood et al. after accounting for race, age and educational level [88]. Another American study analysed a cohort of 1153 low-income minority pregnant women and reported a significantly higher risk of caesarean section in women with vitamin D concentrations lower than 30 nmol/L between the 8th and the 18th week of pregnancy [87]. Other researchers found no association between the first trimester vitamin D concentration and the risk of caesarean section [68,89]. A British study separately analysed the indications for elective and emergency caesarean section [90]. After accounting for cofactors such as BMI, ethnicity and age, the authors found no differences in vitamin D concentrations measured between the 11th and the 13th week of pregnancy in women who delivered vaginally, had elective or emergency caesarean section [90].

A meta-analysis of randomized controlled trials found no effect vitamin D supplementation on the risk of caesarean section [91].

The wide variety of indications for elective and emergency caesarean section and the methodological variability among studies pose a crucial limitation to more adequate evaluation of the effect of vitamin D on the occurrence of caesarean section. Thus, there is at present insufficient evidence about the association of vitamin D deficiency during pregnancy and the risk of caesarean section. Vitamin D supplementation with the aim of reducing the incidence of caesarean section is currently not indicated.

## 8. Conclusions

The aim of this review was to present the findings of different kinds of studies on vitamin D and pregnancy complications. As presented, each type of study has strengths and limitations. Meta-analyses of observational studies often show an association between vitamin D deficiency and pregnancy complications; however, the possibility of important confounding bias must be considered On the other hand, several randomised controlled trials failed to prove an association between vitamin D supplementation and reduction of the risk of various complications, which could be due to the inadequate power or other limitations of the studies, but it could also mean that the observed associations are in fact not causative.

Despite these problems of interpretation, recent evidence suggests that vitamin D supplementation could be of value in reducing the risk of pregnancy complications such as gestational diabetes, preeclampsia, and preterm labour. Vitamin D supplementation in pregnancy has been clearly shown to be safe and can thus be recommended given its potential benefits. New high quality clinical studies and meta-analyses of high quality data are still needed to throw further light on the subject. 
